# Contrast-Free Super-Resolution Power Doppler (CS-PD) Based on Deep Neural Networks

**DOI:** 10.1109/TUFFC.2023.3304527

**Published:** 2023-10-17

**Authors:** Qi You, Matthew R. Lowerison, Yirang Shin, Xi Chen, Nathiya Vaithiyalingam Chandra Sekaran, Zhijie Dong, Daniel Adolfo Llano, Mark A. Anastasio, Pengfei Song

**Affiliations:** Department of Bioengineering, Beckman Institute for Advanced Science and Technology, University of Illinois Urbana–Champaign, Urbana, IL 61820 USA; Department of Electrical and Computer Engineering, Beckman Institute for Advanced Science and Technology, University of Illinois Urbana–Champaign, Urbana, IL 61820 USA; Department of Electrical and Computer Engineering, Beckman Institute for Advanced Science and Technology, University of Illinois Urbana–Champaign, Urbana, IL 61820 USA; Department of Electrical and Computer Engineering, Beckman Institute for Advanced Science and Technology, University of Illinois Urbana–Champaign, Urbana, IL 61820 USA; School of Molecular and Cellular Biology, Beckman Institute for Advanced Science and Technology, University of Illinois Urbana–Champaign, Urbana, IL 61820 USA; Department of Electrical and Computer Engineering, Beckman Institute for Advanced Science and Technology, University of Illinois Urbana–Champaign, Urbana, IL 61820 USA; School of Molecular and Cellular Biology, Beckman Institute for Advanced Science and Technology, University of Illinois Urbana–Champaign, Urbana, IL 61820 USA; Department of Bioengineering, Beckman Institute for Advanced Science and Technology, University of Illinois Urbana–Champaign, Urbana, IL 61820 USA; Department of Electrical and Computer Engineering and the Department of Bioengineering, Beckman Institute for Advanced Science and Technology, University of Illinois Urbana–Champaign, Urbana, IL 61820 USA

**Keywords:** Contrast-free, deep neural networks, microvessel imaging, power Doppler, super-resolution, ultrasound

## Abstract

Super-resolution ultrasound microvessel imaging based on ultrasound localization microscopy (ULM) is an emerging imaging modality that is capable of resolving micrometer-scaled vessels deep into tissue. In practice, ULM is limited by the need for contrast injection, long data acquisition, and computationally expensive postprocessing times. In this study, we present a contrast-free super-resolution power Doppler (CS-PD) technique that uses deep networks to achieve super-resolution with short data acquisition. The training dataset is comprised of spatiotemporal ultrafast ultrasound signals acquired from in vivo mouse brains, while the testing dataset includes in vivo mouse brain, chicken embryo chorioallantoic membrane (CAM), and healthy human subjects. The in vivo mouse imaging studies demonstrate that CS-PD could achieve an approximate twofold improvement in spatial resolution when compared with conventional power Doppler. In addition, the microvascular images generated by CS-PD showed good agreement with the corresponding ULM images as indicated by a structural similarity index of 0.7837 and a peak signal-to-noise ratio (PSNR) of 25.52. Moreover, CS-PD was able to preserve the temporal profile of the blood flow (e.g., pulsatility) that is similar to conventional power Doppler. Finally, the generalizability of CS-PD was demonstrated on testing data of different tissues using different imaging settings. The fast inference time of the proposed deep neural network also allows CS-PD to be implemented for real-time imaging. These features of CS-PD offer a practical, fast, and robust microvascular imaging solution for many preclinical and clinical applications of Doppler ultrasound.

## Graphical Abstract

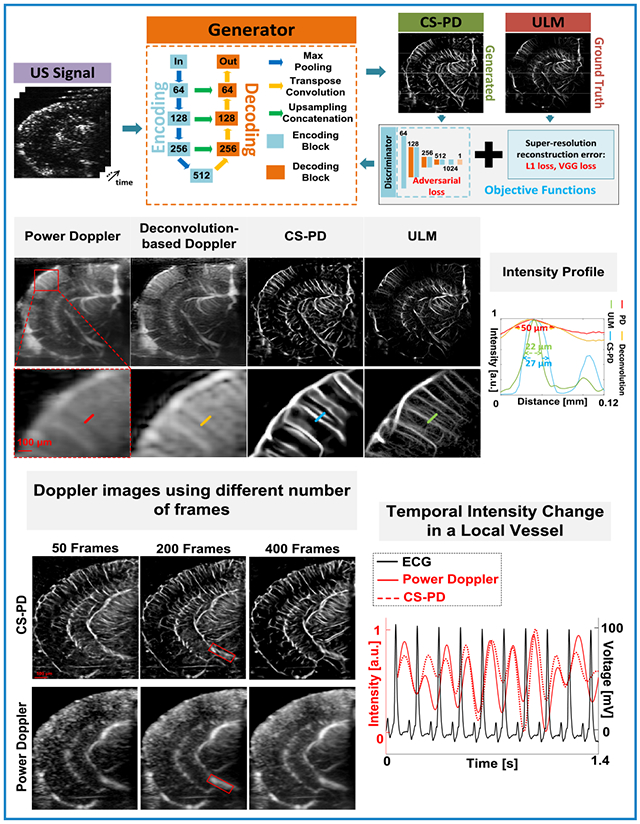

## I. Introduction

SUPER-RESOLUTION ultrasound microvessel imaging is a rapidly growing field. Early studies conducted by Viessmann et al. [1], Desailly et al. [2], [3], and O’Reilly and Hynynen [4] showed that one can break the resolution limit of acoustic waves by localizing microbubbles (MBs) in the blood stream. The seminal papers by Errico et al. [5] and Christensen-Jeffries et al. [6] catalyzed the growth of the field, leading to many subsequent reports with successful in vivo applications [7], [8], [9], [10], [11], [12], [13], [14], [15], [16] and technical advancements of super-resolution ultrasound imaging [17], [18], [19], [20], [21], [22], [23], [24], [25], [26], [27], [28], [29], [30], [31], [32].

At present, most super-resolution ultrasound imaging techniques rely on MB localization, e.g., ultrasound localization microscopy (ULM) [6]. However, these techniques are challenged by the competing goals of minimizing data acquisition time and reconstructing microvessel images that are complete [33], [34], [35]. For instance, to reconstruct the 2-D cross section of a vessel with a diameter of 100 *μ*m, one needs at least 50 independent MB events occurring at unique spatial locations (assuming a uniform MB diameter of 2 *μ*m). However, in practice, this is a challenging task because MB events are dictated by local MB concentration and blood flow and therefore entirely stochastic. Consequently, a long data acquisition time is typically required to accumulate adequate MB signals to fully reconstruct the tissue vasculature. As such, the temporal resolution of ULM is inherently limited by the nature of MB flow in the blood stream and the MB localization process. Another pragmatic challenge of ULM is that a relatively stable MB concentration is necessary to ensure optimal image quality. However, MB concentration is difficult to control in practice, especially with bolus injections that are commonly used in preclinical and clinical practices. Therefore, the objective of this study was to explore an alternative solution for super-resolution microvascular imaging without the need of contrast MBs or the localization process.

In the past decade, a substantial body of literature has reported the combination of long Doppler ensembles with advanced clutter filters [e.g., the ones based on singular value decomposition (SVD)] to increase Doppler sensitivity to small vessels [36], [37], [38]. In addition to improve Doppler sensitivity (e.g., SNR), several methods were recently proposed to improve the spatial resolution of contrast-free power Doppler [39], [40], [41], [42]. For example, Bar-Zion et al. [39] used hundreds of narrowband filters to slice the Doppler signal in the temporal dimension and performed sparsity-based reconstruction on individual signal slices. This technique demonstrates improved spatial resolution but still requires relatively long data acquisition to be effective; Lok et al. [40] used deconvolution with total variation regularization to improve the spatial resolution of contrast-free microvascular imaging. This technique does not require long data acquisition time and is computationally efficient. However, the deconvolution technique requires *a priori* knowledge of the point spread function (PSF) and noise distribution of the ultrasound system, which are difficult to obtain in practice. Jensen et al. [41] and Park et al. [42] reconstructed microvessel images by localizing speckle patterns of red blood cells (RBCs). However, the accuracy and efficiency RBC localization may be compromised by the dense distribution of RBCs in the blood flow.

Super-resolving the tissue microvasculature using backscattering signals from RBCs is challenging because RBC signals are much weaker and denser than the MB signals. Consequently, using native RBCs for super-resolution imaging becomes a challenging problem. Inspired by the recent advances of super-resolution image reconstruction based on deep neural networks in various biomedical imaging modalities [43], [44], [45], [46], [47], in this work, we propose to use deep learning to achieve contrast-free super-resolution microvessel imaging based on RBCs for ultrasound. Our hypothesis is that deep neural networks are capable of 1) learning the distributions of contrast-free ultrasound data and ULM images and 2) translating contrast-free ultrasound data into super-resolved microvascular images by minimizing the distance between these two distributions. However, different from conventional deep learning-based super-resolution image reconstruction techniques where pairs of stationary low-resolution and high-resolution images are used for training, we propose to use spatiotemporal ultrasound data for training. The motivation for using spatiotemporal data comes from observations that single image super-resolution reconstruction techniques are susceptible to hallucinations [48], [49], which is a major concern for biomedical imaging. Our hypothesis is based on the super-resolution optical fluctuation imaging technique (SOFI) [50], where it was demonstrated that imaging resolution can be improved by leveraging the high-order statistics of the fluctuating signal in the temporal dimension. A similar technique [21] has also been applied in the ultrasound field, which achieved an enhanced spatiotemporal resolution for microvascular imaging. In this article, we designed a U-Net-structured deep neural network that generates super-resolution microvessel images from spatiotemporal contrast-free ultrasound signal. A variety of loss functions including the conventional L1 loss, the feature space loss, and the adversarial loss were integrated and investigated in the proposed neural network.

The remainder of this article is organized as follows. In Section II, we present the mathematic model of the proposed super-resolution microvascular reconstruction technique, followed by the deep neural network training strategy. In Section III, the results from the in vivo mouse brain study, in vivo chicken embryo chorioallantoic membrane (CAM) study, and in vivo human study are presented. In Sections IV and V, we finalize this article with discussion and conclusions.

## II. Method

### A. Principles of CS-PD

The 3-D ultrasound signal subject to the SVD clutter filter [23] can be expressed as

(1) $$U(x,z,t)=\sum_{n=1}^{N(t)}H_{n}(t)*P(x_{n}(t),z_{n}(t))+e(x,z,t)$$

where U(x,z,t) represents the spatiotemporal ultrasound signal (in 2-D imaging) with (x,z,t) denoting lateral, axial, and temporal dimension, respectively; n is an index for the scatterers in the blood flow (e.g., index of MBs in contrast-enhanced ultrasound or index of RBCs in contrast-free imaging); N(t) is the total number of scatterers at time t; $H_{n}(t)$ represents of the impulse response of the nth scatterer at time t; $P_{n}(x_{n}(t),z_{n}(t))$, represents the ultrasound imaging system PSF at location $x_{n}(t)$ and $z_{n}(t)$, where $x_{n}(t)$ and $z_{n}(t)$ are the lateral and axial locations of the nth scatterer at time t, respectively; and e(x,z,t) represents the electronic noise at time t.

In localization-based super-resolution microvascular imaging, the task is to recover the locations of the scatterers, $\left(x_{n}(t),z_{n}(t)\right)$, followed by the accumulation of locations to reconstruct super-resolved microvessel images S(x,z)

(2) $$S(x,z)=\sum_{t=1}^{T}\sum_{n=1}^{N(t)}\delta (x-x_{n}(t),z-z_{n}(t))$$

where T represents the total imaging time and δ represents the Dirac delta function. In ULM, the task of obtaining S(x,z) is achieved by various MB localization algorithms [5], [6], [7], [23].

For the proposed contrast-free super-resolution reconstruction, we use a deep neural network f to approximate S(x,z)

(3) f[U(x,z,t);θ]=S(x,z)

where θ represents the parameters of the neural network, which are estimated by minimizing a loss function L. The minimization of the loss function is calculated using the M training data, where M denotes the number of training data pairs that include the spatiotemporal ultrasound signal U(x,z,t) and its corresponding ULM image

(4) $$\hat{\theta }=\underset{\theta }{\text{argmin}}\sum_{i=1}^{M}L\{f[U_{i}(x,z,t);\theta ],S_{i}(x,z)\}.$$

After training, the network f learns the translation from the original ultrasound signal U(x,z,t) to the super-resolved vessel structure S(x,z). L{⋅} represents the objective function for calculating the training loss.

In this study, we have adopted several complementary loss functions, including the L1 loss, the feature space loss [51], and the adversarial loss [52]. These loss terms have been widely used in a variety of super-resolution image reconstruction applications, including ULM, magnetic resonance imaging (MRI), optical and photoacoustic microscopy, and computer vision [22], [43], [44], [45], [46], [47]. The L1 loss promotes the underlying spatial sparsity within the microvascular structure [22] but struggles to recover high-frequency details [45], which can be complemented by the adversarial loss that minimizes the distance of the low-dimensional manifold between the two data distributions [45], [46]. We also incorporated the feature space loss that minimizes the Euclidean distance between the feature maps extracted from the VGG19 network [51], which improves the perceptual quality of the recovered super-resolution images [45], [46], [53]. In this study, three different objective functions were used

(5) $$L\{\cdot \}=L_{1}\{\cdot \}\;L\{\cdot \}=L_{1}\{\cdot \}+\lambda_{1}L_{\text{VGG}}\{\cdot \}\;L\{\cdot \}=L_{1}\{\cdot \}+\lambda_{2}L_{\text{adversarial}}\{\cdot \}$$

where $L_{1}\{\cdot \}$, $L_{\text{VGG}}\{\cdot \}$, and $L_{\text{adversarial}}\{\cdot \}$ represent the L1 loss, the feature space loss, and the adversarial loss, respectively; $\lambda_{1}$ and $\lambda_{2}$ are the regularization parameters, whose values were empirically determined as $\lambda_{1}=0.001$ and $\lambda_{2}=0.01$ to allow contrast-free super-resolution power Doppler (CS-PD) to achieve the visually optimal reconstructed images. The performance of different loss terms was systematically tested for the proposed CS-PD technique.

### B. Deep Neural Network Design

**Fig. 1.**
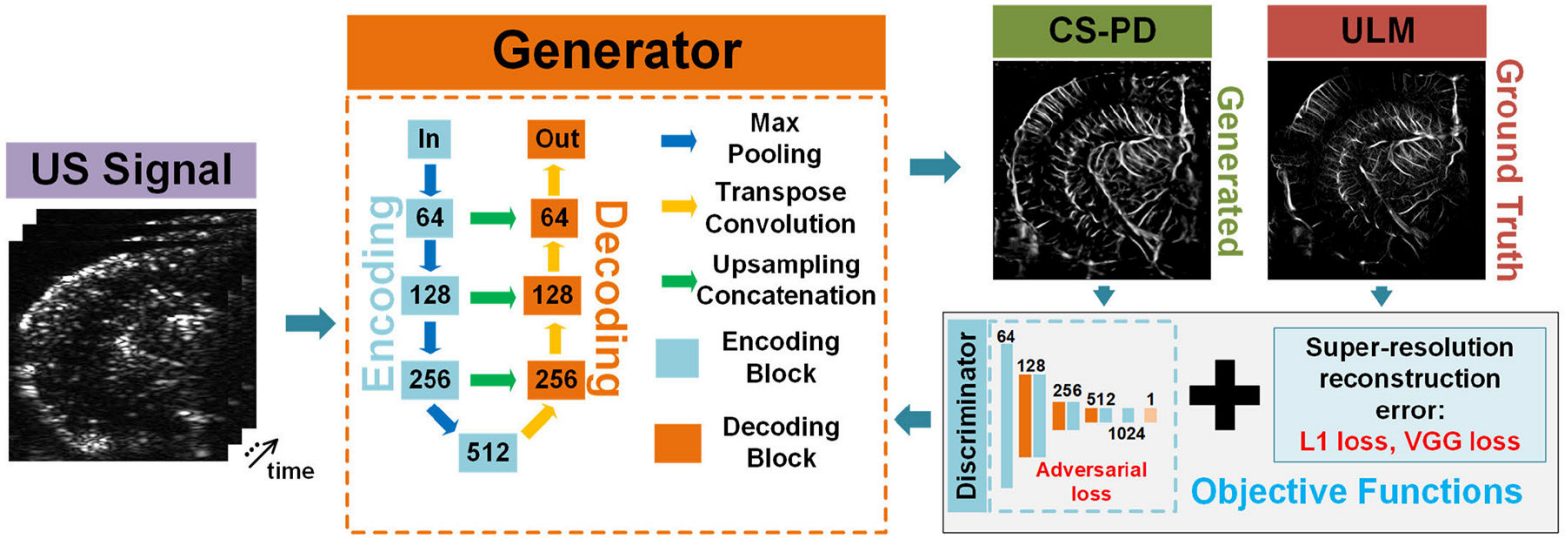
Architecture of the proposed deep neural network. The generator takes the spatiotemporal ultrasound signal as an input and outputs an estimate of the CS-PD image. The generated CS-PD image is compared with the ULM ground truth image to calculate the training loss through the objective function, which was then backpropagated to the generator. The numbers marked in the generator and discriminator represent the number of channels for the convolutional layers within the respective network.

Fig. 1 shows the schematic of the designed deep neural network for CS-PD. The input to the neural network is the spatiotemporal ultrasound signal U(x,z,t) after clutter filtering. The generator network represents the deep neural network function f used for reconstructing the CS-PD image from the input ultrasound signal. The corresponding ULM image represents the ground truth and was used for calculating the training loss using the objective function L. The training loss was then backpropagated to the generator to adjust the network parameters using the Adam gradient descent algorithm [54].

The generator was designed using a U-Net encoding–decoding architecture. In the encoding part, each encoding block consists of two 3 × 3 convolutional layers with batch normalization (BN) and rectified linear unit (ReLU) activations, followed by a 2 × 2 max pooling layer to downsample the image. In the decoding part, each decoding block includes a 2 × 2 transpose convolutional layer with a stride of 2 and two 3 × 3 convolutional layers with BN and ReLU. An additional 2 × 2 bilinear upsampling layer was added after each decoding block to increase the image size. The concatenation between encoding and decoding blocks also includes a bilinear upsampling layer to match the size of the images between encoding and decoding blocks.

To calculate the adversarial loss, a discriminator network was used to output a probability for distinguishing the generated CS-PD image and the ULM ground truth. The discriminator network [45] includes one input block, one output block, and three downsampling blocks. The input and output blocks are composed of a 3 × 3 convolutional layer. The downsampling blocks include two 3 × 3 convolutional layers, where the latter one uses a stride of 2 to downsample the image. All the convolutional layers in the discriminator used leaky ReLU activation with a negative slope of 0.2. The discriminator and the generator were trained simultaneously by minimizing the approximation of the Earth-Mover (EM) distance [55].

### C. Training and Testing Methods

In vivo data from mouse brain was used in this study for training and testing. All mouse brain data were acquired using a Verasonics Vantage 256 System (Verasonics, Kirkland, WA, USA) and an L35-16vX transducer (Verasonics, Kirkland, WA, USA). In addition to the mouse brain, chicken embryo CAM data were acquired with the Verasonics system and the L35-16vX transducer. Imaging was performed using plane-wave compounding with steering angles from −4° to 4° with a step size of 1° and with a transmit frequency of 20 MHz and signal length of 2 cycles. The postcompounding frame rate was 1000 Hz. Electrocardiogram (ECG) signals of the mouse were acquired using an iWorx (Dover, NH, USA) IA-100B single-channel biopotential amplifier with C-MXLR-PN3 platinum needle electrodes inserted into the limbs of the mouse. The details of the animal procedure were provided in our previous studies [12], [34].

For the in vivo human data, the kidney and liver from a healthy volunteer were used in this study. The data are identical to the ones used in [38], which was acquired using an L11-4v transducer (Verasonics Inc., Kirkland, WA, USA) operating at 5 MHz with plane-wave compounding from −9° to 9° with a step size of 2° at a postcompounding frame rate of 500 Hz. The human data were only used for testing.

The ultrasound signal was processed using the SVD clutter filter [38] and the flow separation filters [25] and then input into the deep neural network to generate the CS-PD images. The ULM images were reconstructed using a Kalman filter-based localization and tracking algorithm [23], [24]. All the animal experiments in this study were approved by the Institutional Animal Care and Use Committee (IACUC) at the University of Illinois Urbana–Champaign, Urbana, IL, USA.

The design of the training, validation, and testing dataset was listed in Table I. We acquired the ultrasound data from 51 different image planes in 17 mouse brains (three imaging planes from each mouse). For robust network training with large datasets, both contrast-enhanced and contrast-free ultrasound data were used for training. This study design was justified by the similar flowing pattern and data characteristics of the RBC and MB signals in the blood stream. Specifically, nine blocks of 400 frames of contrast-enhanced ultrasound data and one block of 400 frames of contrast-free ultrasound data were acquired for each imaging plane as the network input. One ULM image reconstructed using 32 000 frames was used as the ground truth. The training dataset was augmented by a factor of 8 using the flow separation filter [25], where 10% of the contrast-enhanced and contrast-free training dataset were randomly selected as the validation dataset ahead of the training process. The validation dataset was used for a learning rate scheduler, which initialized with a learning rate of 0.001 and decayed by a factor of 0.1 when the validation loss stopped improving for five epochs. The number of epochs used for training was set to 50. For testing, we acquired ultrasound data from six imaging planes from two additional mice (three imaging planes from each mouse), whose data were never used for training. One block of 400 frames of contrast-enhanced and contrast-free ultrasound data from each imaging plane was used as the network input, and one ULM image reconstructed using 32 000 frames was used as the ground truth. Finally, 400 frames of contrast-free ultrasound data from a chicken embryo CAM, a human liver, and a human kidney were acquired to test the generalization ability of the proposed network.

### D. CS-PD Performance Evaluation

The imaging performance of the proposed CS-PD technique was assessed by calculating the ensemble-averaged estimates of the peak signal-to-noise ratio (PSNR) and the structural similarity index measure (SSIM) using all the data in the testing dataset. The PSNR and SSIM are defined by [56]

(6) $$\text{PSNR}=-10\cdot \log_{10}[\text{MSE}(a,b)]$$

(7) $$\text{SSIM}=\frac{\left(2\mu_{a}\mu_{b}+c_{1})(2\sigma_{ab}+c_{2}\right)}{\left(\mu_{a}^{2}+\mu_{b}^{2}+c_{1})(\sigma_{a}^{2}+\sigma_{b}^{2}+c_{2}\right)}$$

where a and b represent the normalized generated image and the ground truth, respectively; MSE(·) represents the mean squared error; $\mu_{a}$ and $\mu_{b}$ represent the average intensity of a and b after applying a 3 × 3 Gaussian window; $\sigma_{a}$, $\sigma_{b}$, and $\sigma_{ab}$ represent the variance of a and b and the covariance of a and b after applying the 3 × 3 Gaussian window; c_1_ and c_2_ are two small constants to avoid instability when the denominator is close to zero. PSNR and SSIM were used to evaluate the pixelwise and structural differences between the generated image and the ground truth.

In addition to PSNR and SSIM, we used full-width at half-maximum (FWHM) and MSE of the normalized intensity profile (MSE-NIP) as the specific measurements for the individual vessels. The FWHM was measured on the cross-sectional profile of local vessels to evaluate the resolution improvement of CS-PD. MSE-NIP was measured on the longitudinal profile of the local vessels to evaluate the consistency of the vessel intensity distribution between the CS-PD and ULM. In addition, we compared the frequency components of conventional power Doppler, CS-PD, and ULM images in the Fourier domain [59]. The intersection of the iso-frequency curve and the 1−λ line was used for measuring the resolution. The iso-frequency curve was obtained by calculating the mean value of the frequency contents lying on the iso-frequency ring. An exponential curve fitting was applied on the iso-frequency curve before measuring the spatial frequency. The 1−λ was identified by measuring the spectral amplitude of the power Doppler image at a spatial frequency of one wavelength. The same spectral amplitude was then used to measure the spatial frequencies of ULM and CS-PD images to determine the spatial resolution.

## III. Result

### A. Significance of Temporal Information

**Fig. 2.**
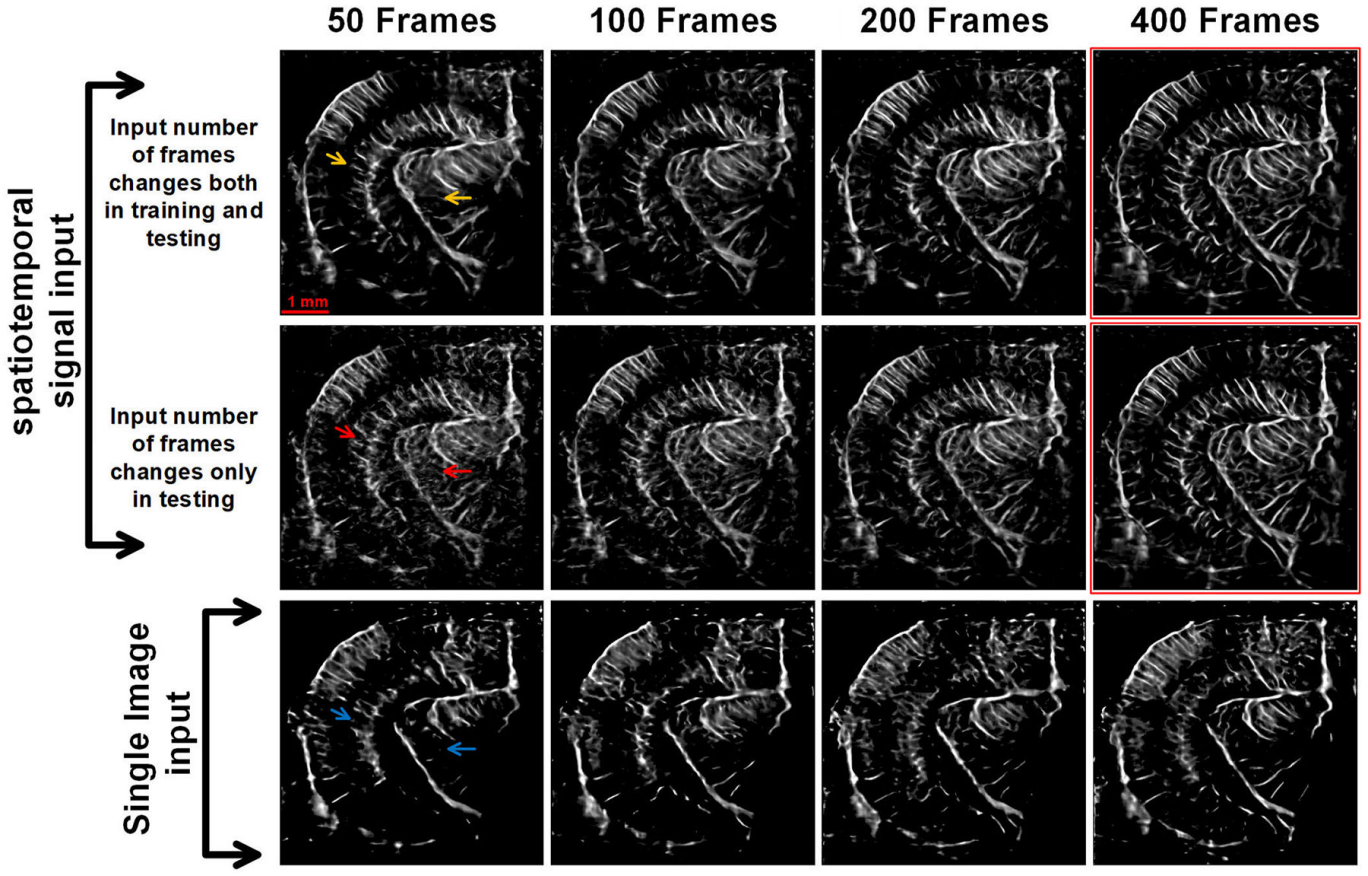
CS-PD using different ways to input ultrasound signal: input number of frames changing both in the network training and testing (first row), input number of frames changing only in the network testing (second row), and inputting power Doppler image after accumulating the spatiotemporal ultrasound signal (third row).

Fig. 2 compares the CS-PD results using different implementations of spatiotemporal ultrasound signal as an input. All CS-PD images and corresponding power Doppler images in Fig. 2 were reconstructed using the contrast-free data acquired from a mouse brain selected from the testing dataset. Three different input strategies were used and compared: 1) adjusting the number of input frames for both network training and testing; 2) truncating the number of input frames only in testing (i.e., the network was trained using the full 400 frames of ultrasound signal, and the test data input ultrasound signal was truncated to a subset of frames and then linearly interpolated in the temporal dimension to 400 frames to match the network architecture weightings); and 3) using the single 2-D power Doppler image after accumulating the ultrasound signal as the network input for both training and testing.

It can be seen in Fig. 2 that the network cannot recover some of the small vessel structures (indicated by the blue arrows) when using single 2-D power Doppler images as an input (i.e., without temporal information). Moreover, hallucination structures (indicated by the red arrows) were created with low number of input frames and a discrepancy between the number of training and testing input frames. In comparison, when using the same number of frames in both training and testing, CS-PD can more robustly reconstruct the microvessel structures with less hallucinations (indicated by the orange arrows). As the number of input frames increases, more and more temporal blood flow information was included in training and testing, which leads to improved CS-PD image quality with more detailed cerebrovasculature (see the images marked by red outline). Results in Fig. 2 reveal that the inclusion of temporal information is essential for the network to differentiate and resolve microvessels with different flowing characteristics, which are otherwise indiscernible with single 2-D power Doppler images as an input.

### B. Validation of the Performance of CS-PD

**Fig. 3.**
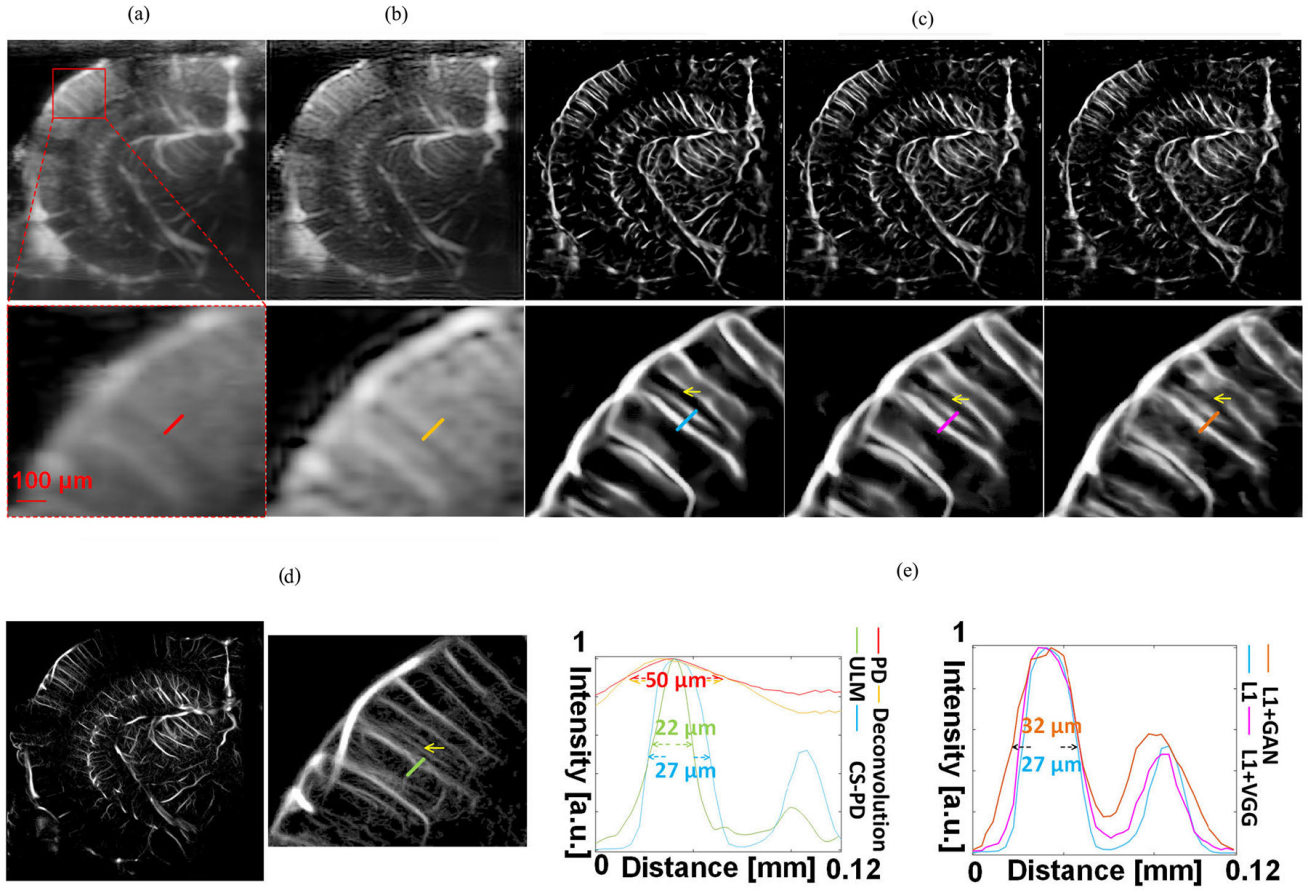
(a) Conventional power Doppler image accumulating 400 frames of contrast-free ultrasound data. (b) Deconvolution-based power Doppler image reconstructed using 400 frames of contrast-free ultrasound data. (c) CS-PD images using different loss functions reconstructed using 400 frames of contrast-free ultrasound data. (d) ULM image reconstructed using 32 000 frames of contrast-enhanced ultrasound data. (e) Intensity profile of the small penetrating cortical vessels marked by the short line segments with different colors. All the intensity profiles were normalized in the linear scale. All the zoomed-in local vessel images (bottom row) correspond to the same region marked by the red box in the power Doppler image (a).

Fig. 3 shows the comparison among conventional Doppler (power Doppler and deconvolution-based Doppler), CS-PD using different loss functions, and the corresponding ULM image. The conventional Doppler images and CS-PD images were reconstructed using the same 0.4 s (400 frames) of contrast-free data from the testing dataset. The corresponding ULM image was reconstructed using 32 s of ultrasound data (corresponding to 32 000 frames) acquired from the same image plane after injecting contrast MBs. The results clearly indicate that CS-PD substantially improved the spatial resolution over conventional Doppler including the deconvolution-based resolution enhancement approach. In the cross-sectional vessel profile measurement [Fig. 3(e), top panel], CS-PD improved the FWHM of a penetrating cortical vessel from 50 to 27 *μ*m, which is similar to the 22-*μ*m FWHM measured from the ULM image.

**Fig. 4.**
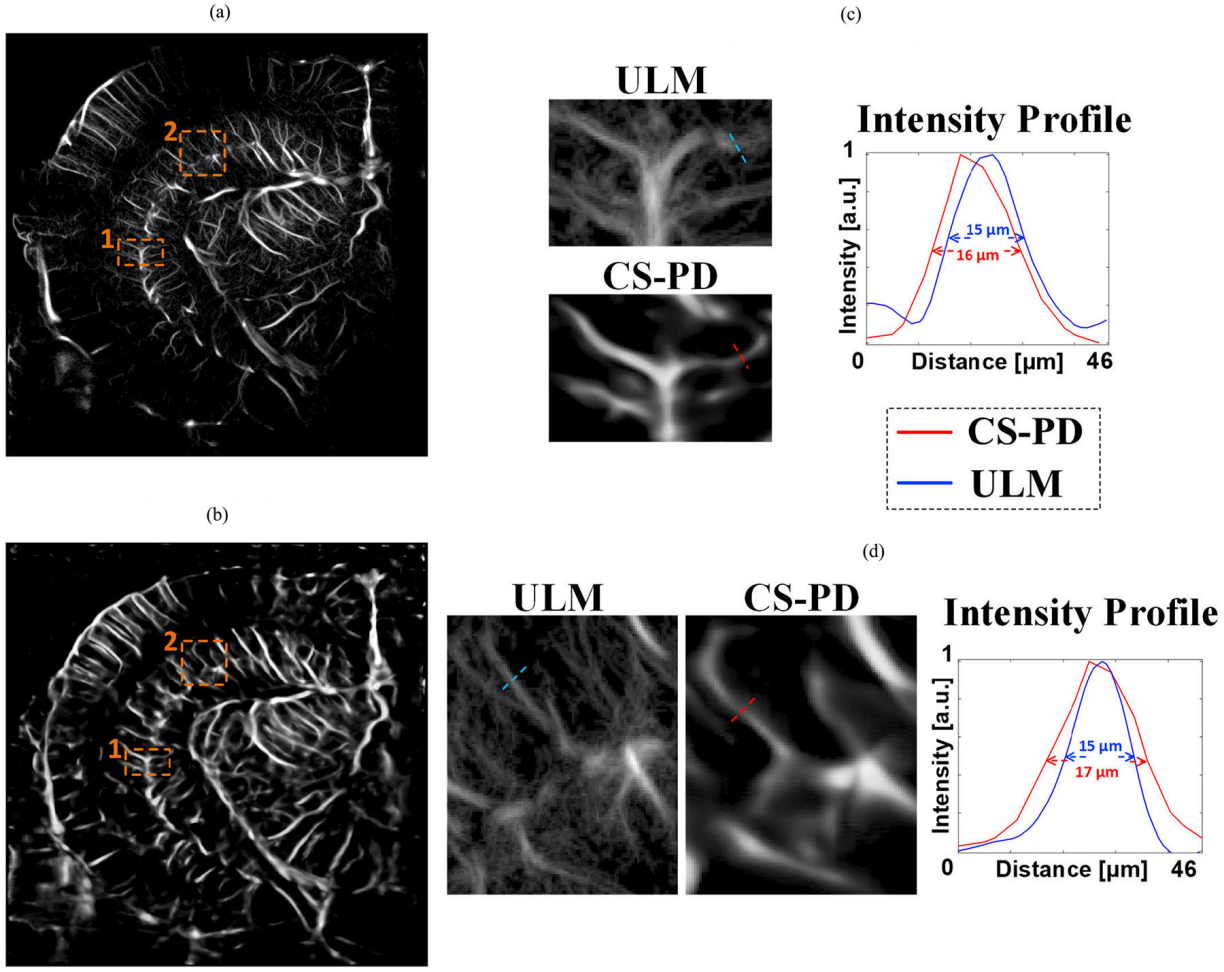
Comparison of (a) ULM and (b) CS-PD. (c) and (d) Magnified local regions as marked by the orange dashed rectangle and the intensity profiles of selected local vessels.

**Fig. 5.**
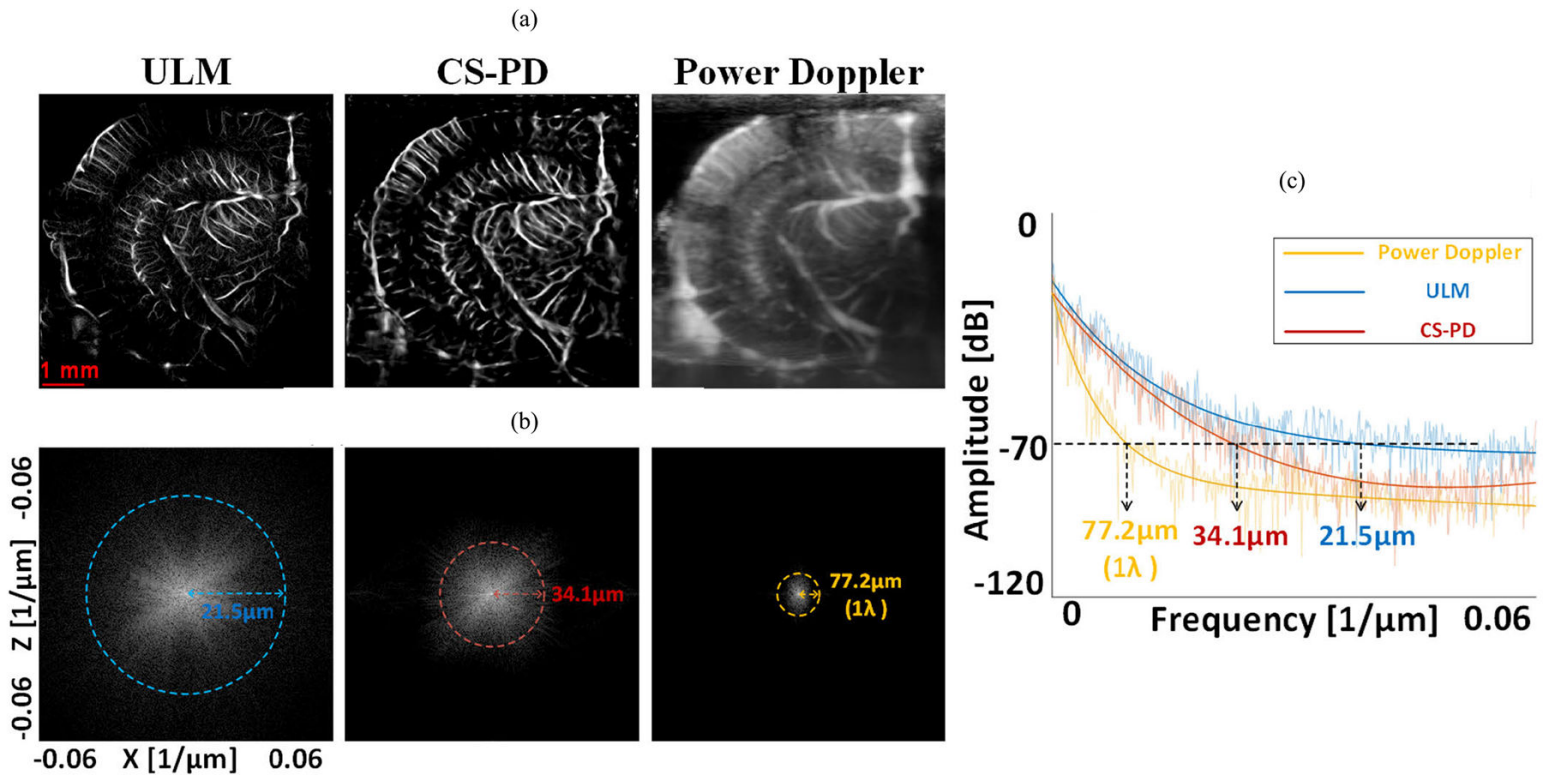
(a) ULM, CS-PD, and conventional power Doppler in the same imaging plane of a mouse brain. (b) Corresponding 2-D Fourier spectra of the images in (a) and the 1−λ iso-frequency rings. (c) Iso-frequency curves of the images in (a) and the corresponding exponential fitting curves. All the images in (b) used 70-dB dynamic range.

We have selected another two smaller vessels in Fig. 3 and compared the intensity profile of CS-PD using L1 loss only and ULM. As shown in Fig. 4, CS-PD achieved a resolution of 16–17 *μ*m for these two vessels that have an FWHM of 15 *μ*m as measured in the ULM image. In addition to that, Fig. 5 shows that the Fourier domain of conventional power Doppler, CS-PD, and ULM showed that CS-PD and ULM achieved the global resolution of 34.1 and 21.5 *μ*m separately when conventional power Doppler has one-wavelength resolution (77.2 *μ*m). Both results indicate that CS-PD can achieve a more than twofold resolution improvement compared with conventional power Doppler.

**Fig. 6.**
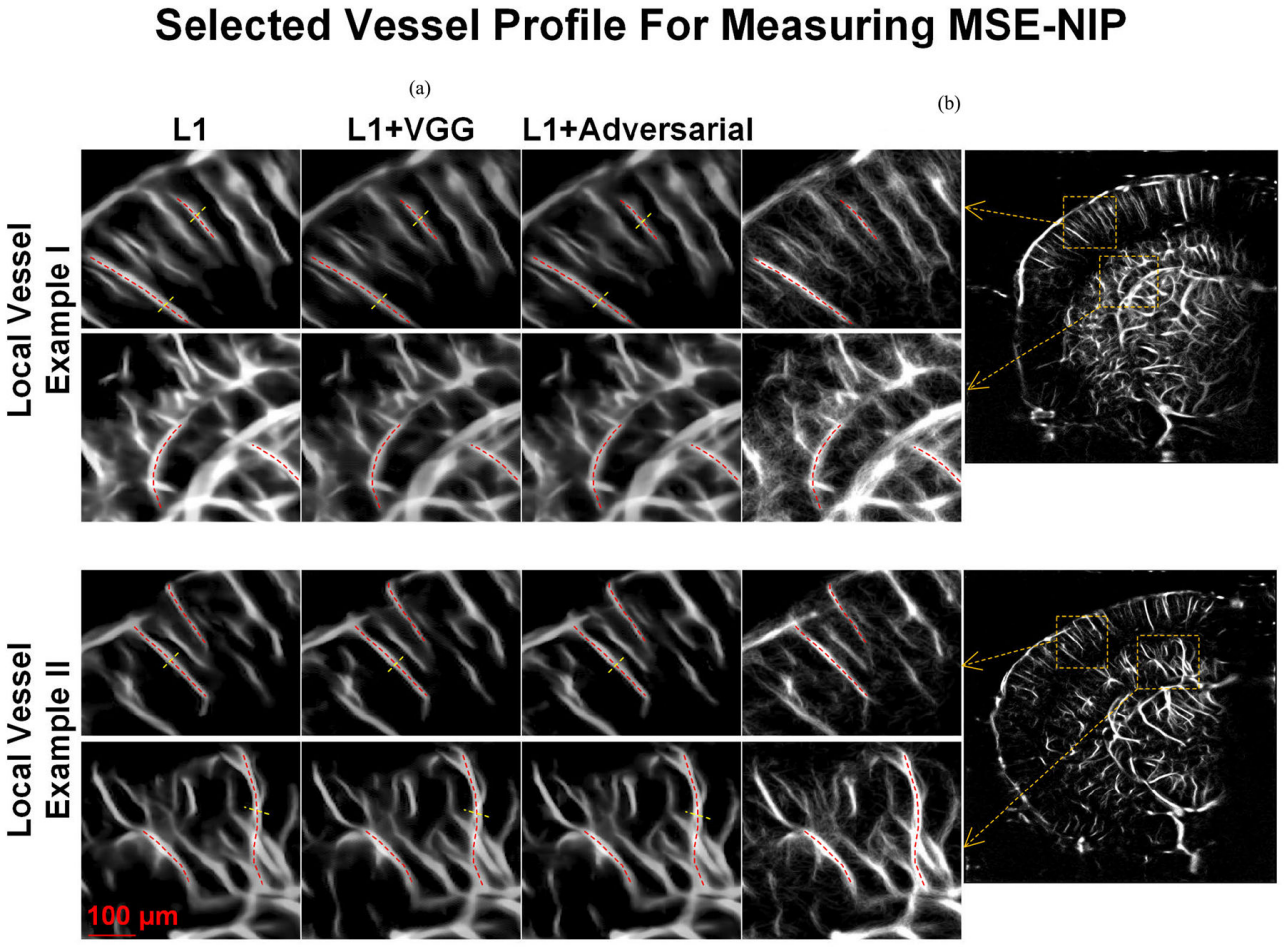
Two examples of the selected vessel profiles for measuring the MSE-NIP (red curves) and FWHM ratio (yellow curves) in Table II. (a) CS-PD using different loss functions. (b) ULM image of the same imaging plane. All the profiles were manually selected along the longitudinal or cross-sectional direction of the vessels.

Table II summarizes the quantitative metrics used to evaluate imaging performance among various techniques. The ensemble-averaged estimates of SSIM and PSNR were evaluated using all the data in the testing dataset. The MSE-NIP and FWHM ratio were evaluated by selecting ten local vessels in the mouse brain images from the testing dataset. The MSE-NIP was measured on the longitudinal profiles of manually selected vessels between CS-PD and ULM images. The FWHM ratio was measured by dividing the FWHM of the cross-sectional profiles of manually selected vessels between power Doppler and CS-PD. Two examples of such selected local vessel profiles are shown in Fig. 6. All the quantitative metrics were measured for the mean and the standard deviation (indicated by the number after ± symbol) across all the samples used for evaluation, as shown in Table II. Among all the CS-PD images, results using L1 with VGG loss and L1 with adversarial loss show more small vessel structures especially for regions that are in between the parallel penetrating vessels [Fig. 3(c)]. However, images generated with L1 loss show the highest SSIM and MSE-NIP measurements (Table II), which indicate the highest consistency with the ULM ground truth. In addition, the vessel profile measured from CS-PD images generated by the L1 loss [Fig. 3(e), right panel] was closest to ULM. Collectively, these results indicate that CS-PD using L1 loss achieved the best performance in terms of SSIM and MSE-NIP but had the worst performance in terms of PSNR and FWHM ratio. As shown in Fig. 3, one reason for the poor PSNR is that the L1 loss missed small vessel structures (indicated by the yellow arrows in Fig. 3) that were recovered by using L1 loss combined with VGG and L1 combined with adversarial loss. However, the low SSIM and MSE-NIP scores from L1 loss combined with VGG or adversarial loss indicate that the recovered small structures may not be valid (i.e., hallucinations) when comparing to ULM. These results imply that due to the lack of contrast enhancement and the subsequent absence of distinct point scatterers like MBs, the small vessel recovery may be fundamentally limited in CS-PD.

### C. Validation of the Temporal Resolution of CS-PD

**Fig. 7.**
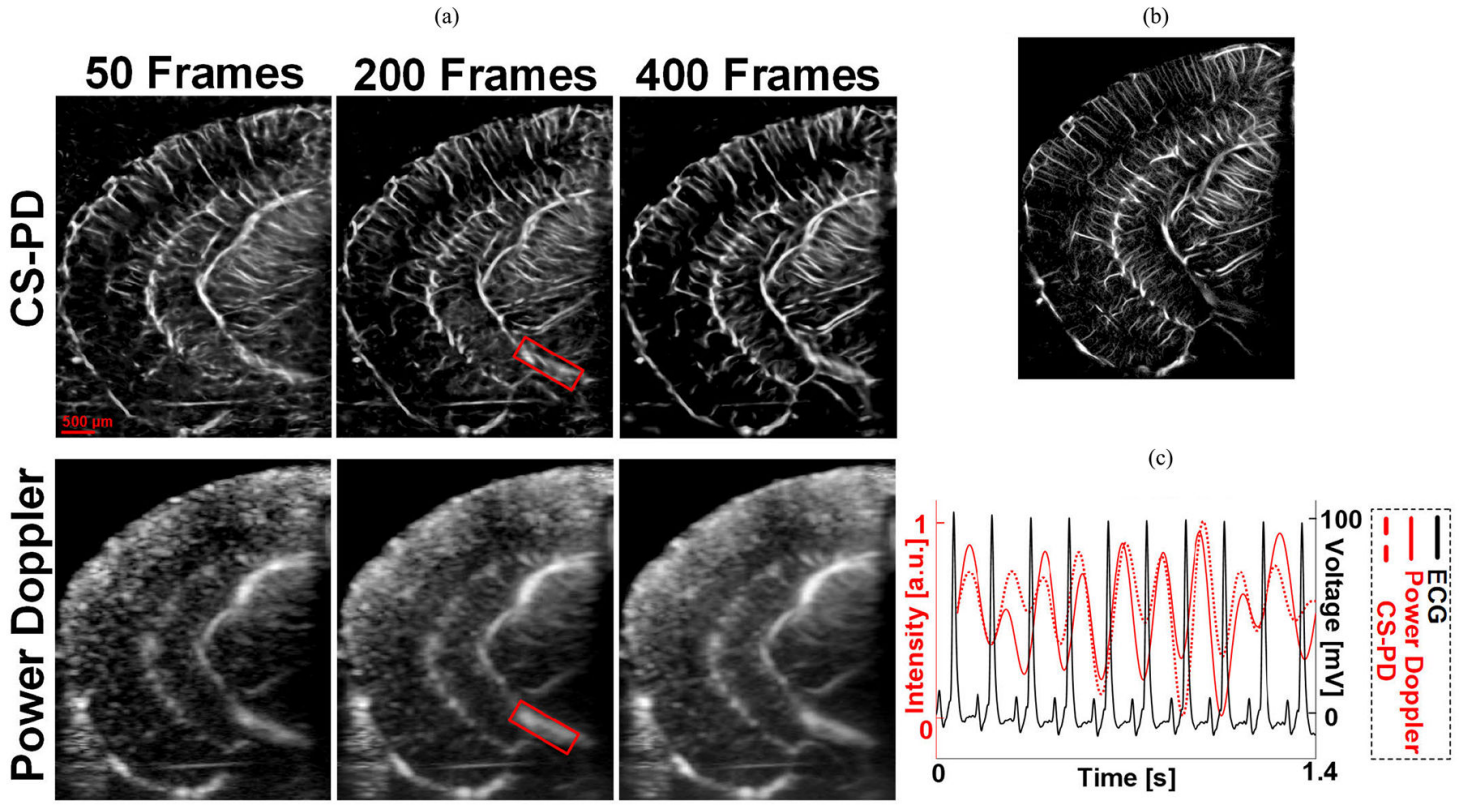
(a) CS-PD and power Doppler images of the same mouse brain using different numbers of frames. (b) ULM image as ground truth. (c) Average intensity curve of the local region marked by the red rectangle in (a) along with the ECG signal. The temporal intensity curve was also demonstrated in the supplementary video 1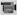.

Fig. 7 shows the comparison of CS-PD and conventional power Doppler using different numbers of frames as an input and the temporal intensity changes of a local vessel. The conventional power Doppler images and CS-PD images were reconstructed using the same contrast-free data from the testing dataset. For CS-PD using less than 400 frames, the number of input frames changed both in the network training and testing. Fig. 7(c) also showcases the ability of preserving temporal information (e.g., pulsatility of the blood flow) for CS-PD. ULM image reconstructed using 32 s of data acquisition (32 000 frames) was used as the ground truth. To extract the temporal blood flow signal, power Doppler and CS-PD were reconstructed using 0.2-s (200 frames) temporal sliding window with a step size of 0.05 s (50 frames). Supplementary video 1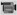 demonstrates the dynamic flow signal revealed by CS-PD and power Doppler. Based on the above results, only L1 loss was used for CS-PD. As shown in Fig. 7(c), the blood flow pulsatility measured from CS-PD showed good agreement with that from conventional Doppler. In addition, both pulsatility measurements showed good agreement with the ECG signal, with the peak of the blood flow occurring after the peak R-wave, which indicates peak systole. These results indicate that CS-PD has similar temporal resolution as conventional power Doppler and is capable of preserving the important temporal information of blood flow, which is significant for applications such as functional brain imaging.

### D. Demonstration of Generalizing CS-PD to Other Tissues Not Included in Training Datasets

**Fig. 8.**
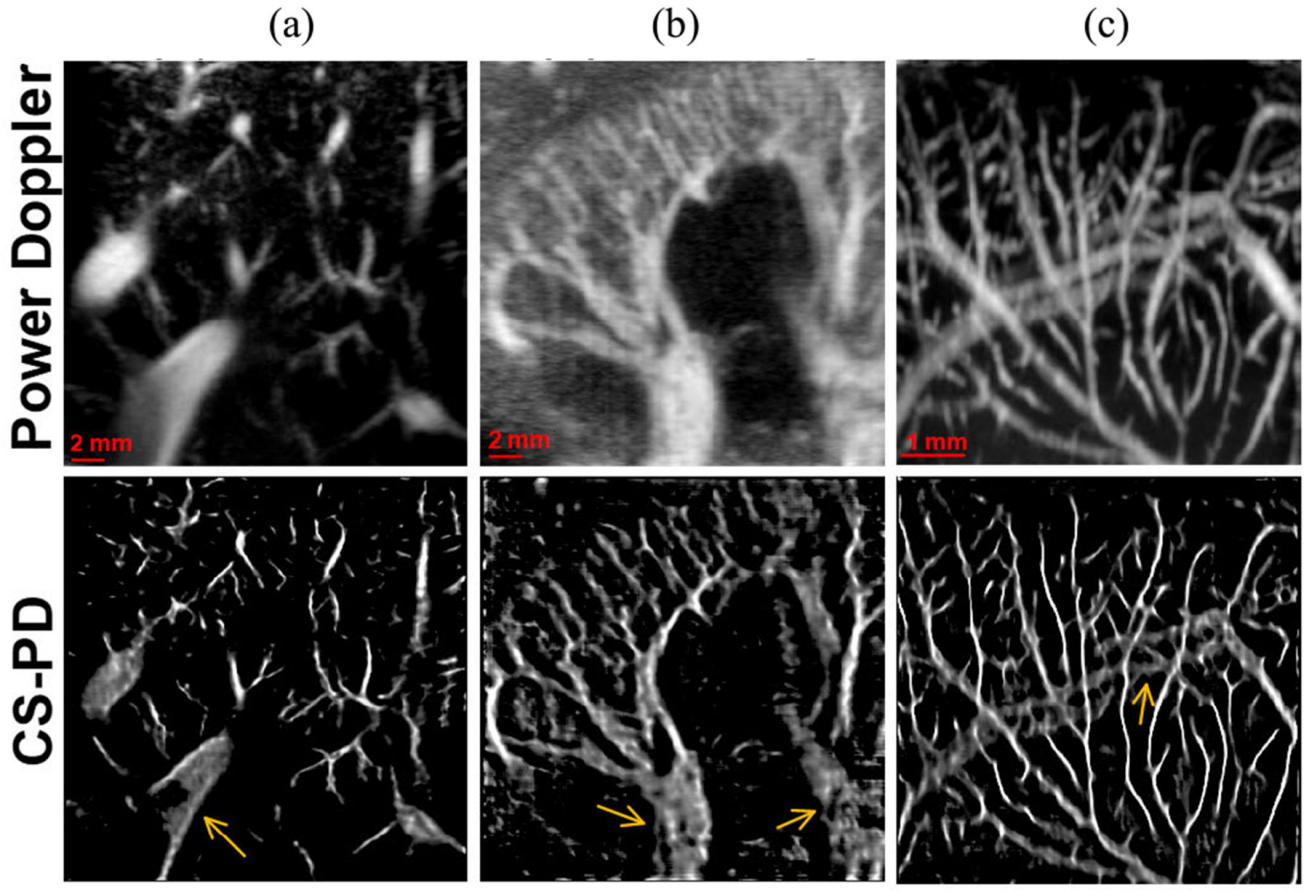
CS-PD and power Doppler images of (a) human liver, (b) human kidney and (c) chicken embryo CAM.

Fig. 8 shows the performance of CS-PD in different types of tissues that were never seen by the deep neural network during training and validation. Both power Doppler and CS-PD images were reconstructed using 0.4 s of contrast-free data acquisition (400 frames). The CS-PD images were reconstructed using the network trained with the L1 loss only. Interestingly, as shown in Fig. 8, CS-PD could reconstruct most of the vessel structures with substantially improved spatial resolution in all the testing cases. This result is not surprising because although different imaging settings (e.g., frequency, transducer) and tissues were used for in vivo human and CAM imaging, the characteristics of the structure for smaller vessels are similar for different tissues. CS-PD did struggle with some of the major vessels that have substantially different size distributions than those in the training dataset (indicated by the orange arrows in Fig. 8). It is expected that CS-PD will gain in performance of reconstructing the larger vessels when data from these larger vessels are included in the training dataset.

## IV. Discussion

This article presented a CS-PD imaging method that uses deep neural networks to reconstruct super-resolved microvessel structure from using spatiotemporal ultrasound signal. The paired ULM images were used as the ground truth during the training, validation, and testing of the proposed neural network. Comprehensive assessments of the performance of CS-PD were provided in this study. First, we compared the performance of CS-PD using either with (e.g., raw spatiotemporal data) or without (e.g., accumulated power Doppler image) temporal information, and the result shows that temporal information was essential for recovering the microvessels with varying flow characteristics (Fig. 2). This finding is consistent with previous studies in optical and ultrasound super-resolution imaging (e.g., SOFI [21], [50]), where temporal blood flow information was utilized to break the diffraction limit of the imaging system.

Second, we used four different quantitative metrics (SSIM, PSNR, FWHM, and MSE-NIP) to validate the performance of CS-PD using different loss functions. Comparison studies with conventional power Doppler and deconvolution-based power Doppler were also conducted. Our results indicate that CS-PD could improve the spatial resolution by an approximate factor of two when compared to conventional Doppler [Fig. 3(d)]. In the study that compared different loss functions, the simplest loss function (e.g., L1 loss) demonstrated the best overall performance as indicated by the highest SSIM and MSE-NIP scores. Although the VGG loss and adversarial loss added more details and small vessel structures that delivered a higher PSNR, they did not provide good agreement with the reference standard ULM image, which indicates that these additional details and smaller vessel structures are more likely to be hallucinations [48], [49]. This result is not surprising because the contrast-free data do not provide adequate information to support the full reconstruction of small vessel structures. CS-PD based on contrast-free ultrasound signal may include additional blood flow information that ULM does not capture. However, for those small vessels that are missed by MB localizations, CS-PD has limited capability to resolve their structures as well. The choice of different loss functions would depend on the specific requirements of the applications that CS-PD is going to be used for. For applications that require accurate measurement of temporal dynamics of the blood flow signal, CS-PD using L1 loss only is a more suitable choice.

**Fig. 9.**
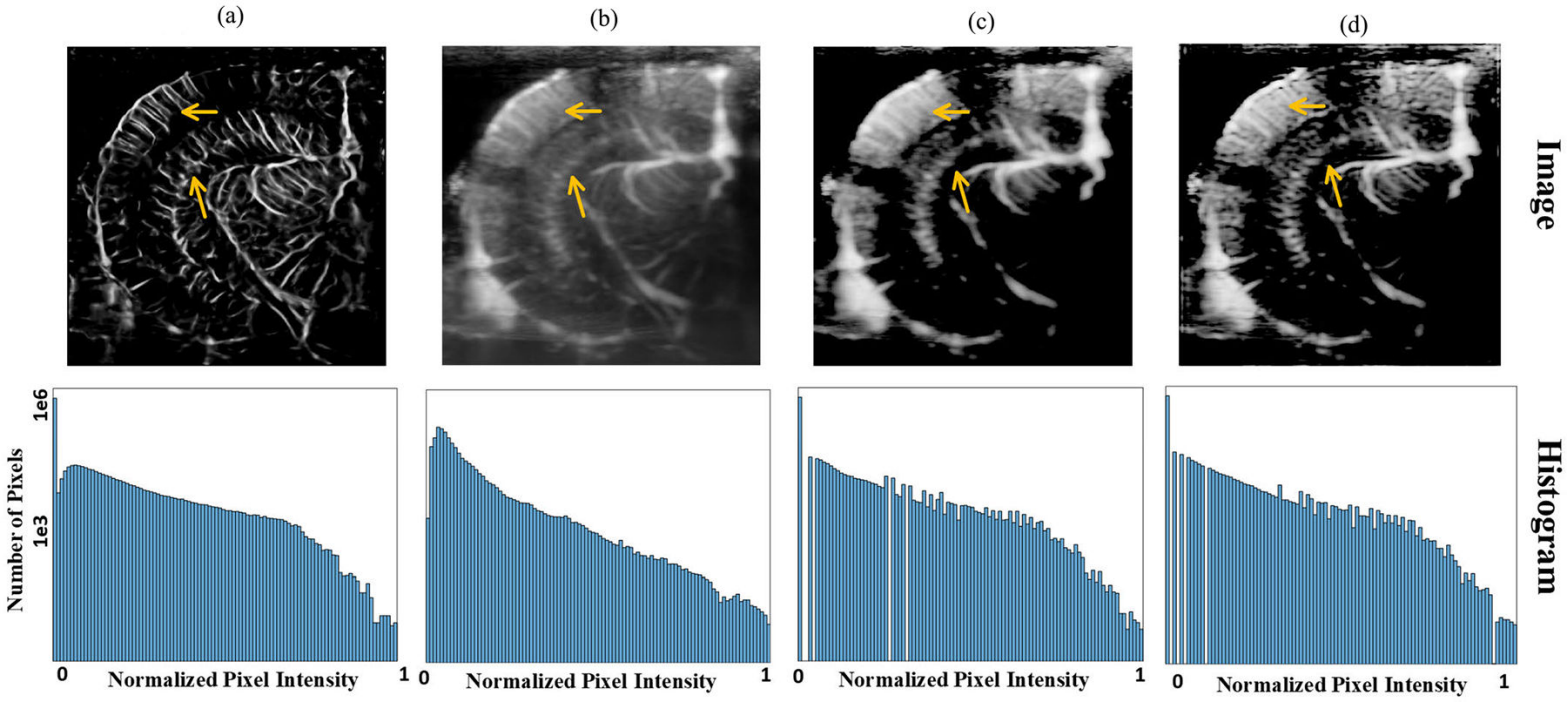
Comparison of the (a) CS-PD, (b) power Doppler, (c) power Doppler after histogram matching, and (d) deconvoluted power Doppler after histogram matching.

Third, we demonstrated that CS-PD could preserve the temporal information of the power Doppler signal when using ECG as the common reference ground truth. As shown in Fig. 7(c), CS-PD presented a temporal blood flow profile that closely resembles the conventional power Doppler signal. This result is significant for applications where the intensity and temporal dynamics of the blood flow signal are important [e.g., functional ultrasound (fUS) imaging]. However, CS-PD is not supposed to have exactly the same amplitude profile as power Doppler since the neural network is not a simple linear system to scale or stretch the input image to different dynamic ranges. This is proved in Fig. 9 where power Doppler and deconvoluted power Doppler after histogram matching cannot achieve the same resolution improvement as CS-PD. This is a limitation of CS-PD when it is used for detecting dynamic blood volume change, because ideally, we want CS-PD to have exactly the same temporal intensity profile as conventional power Doppler. To address this issue, recurrent neural network could be used to extract more temporal intensity information and constrain the temporal difference between CS-PD and power Doppler [32].

Finally, we demonstrated the generalization ability of CS-PD for different tissues that were not included in the training data. Although the training dataset only included ultrasound data acquired from mouse brain, CS-PD still showed robust reconstruction results for human liver, human kidney, and chicken embryo CAM (Fig. 8). This result suggests that CS-PD is not limited to specific vessel structures associated with certain tissue types. As long as the testing data present similar vascular distributions as in the training data, CS-PD will function and provide improved spatial resolution. Our results did indicate suboptimal performance in big vessels that were not included in the training data. To overcome this limitation, larger training datasets with wider vessel data distributions become necessary.

There are some limitations in this study. First, the contrast-enhanced data used for reconstructing the ULM ground truth images were acquired asynchronously with the contrast-free data. A small amount of tissue motion was present between different data acquisitions, which lead to misalignment between the contrast-free and contrast-enhanced data. Although motion correction was implemented [23] to correct for the misregistration that was in-plane, out-of-plane motion remained. This misalignment between the contrast-free data and the ULM ground truth degrades the performance of the trained network. To address this issue, we used a combination of contrast-enhanced and contrast-free dataset for training. The contrast-enhanced data provide a strict correspondence between the flowing scatters and the microvessel structures for the network to learn, while the inclusion of the contrast-free data can adjust the network weights for better characterizing the distribution of the contrast-free dataset. This is a fundamental limitation of the proposed CS-PD technique because it relies on paired ULM images for training. As a consequence, CS-PD achieved suboptimal performance on tissues that were not included in the training dataset (e.g., larger vessels in Fig. 8). To address this issue, accelerated ULM imaging techniques, such as CTSP [26], MUTE [28], deep learning-based techniques [29], [30], [31], [32], and other fast contrast free super-resolution techniques [41], [58], may be considered for fast imaging speed. Nevertheless, based on the robust generalizability of the proposed method, one can also use transfer learning techniques to adapt CS-PD to different types of tissues, where only a small amount of application-specific ULM images is needed to fine-tune the network.

Another limitation of CS-PD is that it is not expected to achieve the same resolution as ULM. As shown in Figs. 3-5, the spatial resolution of CS-PD is not as high as ULM. Although CS-PD uses ULM images as ground truth for training, contrast-free ultrasound signal does not have enough information to support the reconstruction of small vessels that are visible in ULM. For applications that require accurate microvascular morphology, ULM is a more suitable approach. However, compared with ULM, CS-PD has a much higher temporal resolution and does not require contrast injection. These advantages make CS-PD a more pragmatic and convenient imaging approach, where fast imaging and contrast-free are important, which is the case for many preclinical and clinical applications.

The third limitation of CS-PD is that it does not provide the flow direction or speed information as in ULM. Including flow velocity in the network training significantly increases the complexity of the task, which is challenging with the current network structure. Future studies are necessary to explore the possibility of inferring flow speed and direction with the proposed generative network framework.

## V. Conclusion

This article presents a CS-PD imaging method that uses deep neural networks to reconstruct the super-resolved microvessel image from the spatiotemporal ultrasound signal. The performance of the proposed method has been assessed by 1) measuring the quantitative metrics for different loss functions; 2) comparing the temporal intensity profile with conventional power Doppler and ECG signal; and 3) validating the results on different types of tissues beyond the training dataset. The results have shown that CS-PD can robustly reconstruct the microvessel structure using contrast-free ultrasound signal with short acquisitions. This technique provides a practical solution for super-resolution vascular imaging where fast imaging speed is essential.

## Supplementary Material

supp1-3304527

## Figures and Tables

**TABLE I:** Design of the Training, Validation, and Testing Dataset

	Data Acquisition	Network Input	Ground Truth
**Mouse Brain**	**Training**: 51 imaging planes in 17 mice (3 imaging planes each mouse)	10 blocks of 400 frames each imaging plane	1 ULM accumulating 32000 frames each imaging plane
	**Validation**: 51 imaging planes in 17 mice (3 imaging planes each mouse)	Randomly selected 10% of the training dataset	1 ULM accumulating 32000 frames each imaging plane
	**Testing**: 6 imaging planes in 2 mice (3 imaging planes each mouse)	2 block of 400 frames each imaging plane	1 ULM accumulating 32000 frames each imaging plane
**Chicken Embryo CAM/Human liver/Human Kidney**	**Testing**: 1 imaging plane	1 block of 400 frames each imaging plane	

**TABLE II:** Evaluation Metrics of CS-PD Using Different Loss Functions

Loss╲Metrics	SSIM	PSNR (dB)	NIP-MSE	FWHM Ratio (Power Doppler/CS-PD)
**L1**	**0.784**±0.095	24.645±1.650	**0.061**±0.039	2.277±0.593
**L1+VGG**	0.779±0.090	25.105±1.819	0.120±0.057	2.327±0.813
**L1+Adversarial**	0.783±0.090	**25.518**±1.840	0.067±0.024	**2.378**±0.836

## Highlights

- A contrast-free super-resolution power Doppler (CS-PD) imaging method that uses deep neural networks to reconstruct the super-resolved microvessel image from the spatiotemporal ultrasound signal.
- CS-PD could achieve an approximate 2-fold improvement in spatial resolution and the same temporal resolution compared with conventional Power Doppler.
- CS-PD provides a practical solution for super-resolution vascular imaging where fast imaging speed is essential.
